# Cutaneous microvascular reactivity in Charcot neuroarthropathy: a systematic review and meta-analysis

**DOI:** 10.1186/s13047-022-00522-x

**Published:** 2022-03-01

**Authors:** Sean Michael Lanting, Tsz Long Chan, Sarah Louise Casey, Benjamin John Peterson, Vivienne Helaine Chuter

**Affiliations:** 1 College of Health, Medicine and Wellbeing, School of Health Sciences, University of Newcastle, Ourimbah, NSW Australia; 2 Department of Podiatry, School of Health, Medical and Applied Sciences, CQUniversity, Rockhampton, QLD Australia; 3 School of Health Sciences, Western Sydney University, Campbelltown, NSW Australia

**Keywords:** Arthropathy, Neurogenic, Microcirculation, Diabetic neuropathies, Diabetic foot, Diabetes complications

## Abstract

**Background:**

To systematically evaluate the literature investigating the relationship between cutaneous microvascular reactivity in the foot of adults with diabetes-related Charcot neuroarthropathy compared to a non-Charcot adult control group.

**Methods:**

A systematic search was conducted to June 2021 using the biomedical databases EBSCO Megafile Ultimate, Cochrane Library and EMBASE. Original research conducting comparative investigation of cutaneous microvascular reactivity in the foot of adults with diabetes and any pattern of acute or chronic Charcot neuroarthropathy and any non-Charcot adult control groups were included. A modified Critical Appraisal Skills Programme tool was used for quality appraisal. Cutaneous microvascular reactivity in diabetes-related Charcot neuroarthropathy data were synthesised and meta-analysis conducted where possible.

**Results:**

The search strategy identified 1,684 articles, with seven eligible for inclusion. Included studies used various methodologies and equipment to assess cutaneous microvascular reactivity in 553 participants (162 with Charcot neuroarthropathy). Cutaneous microvascular reactivity in Charcot neuroarthropathy groups was impaired compared to uncomplicated diabetes groups. Meta-analysis investigating the difference in response to thermal hyperaemia demonstrated a significant difference in cutaneous microvascular reactivity between Charcot neuroarthropathy and peripheral neuropathy with a large, pooled effect size (SMD 1.46 95% CI: 0.89–2.02) and low heterogeneity (*I*^*2*^ = 4%, *T*^*2*^ *=* 0.01) indicating that the cutaneous microvascular response is more impaired in peripheral neuropathy than in Charcot neuroarthropathy.

**Conclusions:**

Charcot neuroarthropathy is associated with greater cutaneous microvascular reactivity in the periphery relative to diabetes cohorts with diabetes-related peripheral neuropathy alone. It is unknown if this occurs prior to, or as a result of, Charcot neuroarthropathy.

## Background

Charcot neuroarthropathy is a gradual and destructive complication of diabetes mellitus [1] that characteristically affects the bones, joints and tissues of the foot and ankle [2]. The condition is accompanied by increased risk of subsequent foot complications, impaired lower limb function [3, 4], reduced quality of life [5], and premature mortality [6]. Although not comprehensively understood, it is widely accepted that the pathogenesis of Charcot neuroarthropathy involves a combination of neural and vascular dysfunction with fractures and dislocations of the foot often the acute presentation [5]. Though Charcot neuroarthropathy may develop as sequelae of peripheral neuropathy from an array of origins, diabetes is now considered the primary aetiology [2] and has a reported prevalence of between 0.08% and 13% in diabetes foot clinics [7]. With over 450 million people worldwide estimated to have diabetes [8], there is a great need to further understand the genesis of this pathology in order to guide prevention and treatment strategies.

Microvascular dysfunction manifesting as complications including peripheral neuropathy, retinopathy or nephropathy, is a well-documented hallmark of long-standing or poorly controlled diabetes [9]. While the link between diabetes-related peripheral neuropathy (DPN) and Charcot neuroarthropathy is well established, the contribution of other aspects of microvascular dysfunction, particularly relating to localized blood flow regulation in the periphery, is less clear [10]. Two theories have been proposed in relation to the development of Charcot neuroarthropathy. The neurovascular theory suggests that autonomic neuropathy, occurring as a result of sympathetic denervation results in the vasodilation of peripheral vasculature and the development of arteriovenous shunting whereby blood is diverted away from the superficial capillary beds in the skin and increases blood flow to bone [2, 11, 12]. This is suggested to result in an increase in osteoblastic activity, resulting in bony demineralization and weakening, and increased risk of bone trauma [2, 13]. In contrast, the neurotraumatic theory, proposes Charcot neuroarthropathy is a response to undetected, repetitive microtrauma from excessive mechanical stress on bone and joints and a result of neuropathy. Undetected bone trauma and continued weight bearing is suggested to result in an excessive inflammatory response which results in subsequent change in vascular function and bone demineralisation that are characteristic of the condition [2, 12–14].

In contrast to the typical presentation of Charcot neuroarthropathy, diabetes itself and DPN are typically associated with a reduction in microvascular blood flow through both structural changes to capillaries, a reduction in capillary density and neurological impairment to microvascular reactivity resulting in a functional ischaemia in the presence of injury [15]. The proposed theoretical response instigating the development of Charcot neuroarthropathy suggests a fundamental difference in microvascular function in those affected by the condition compared to those with DPN alone. Identifying differences in microvascular function in those with diabetes with, and without, Charcot neuroarthropathy has the potential to provide clinical assessment methods to better identify those at risk of the condition.

The aim of this review was to systematically evaluate the literature comparing the relationship between cutaneous microvascular reactivity in the foot of adults with diabetes-related Charcot neuroarthropathy and other patient phenotypes such as those with diabetes or DPN only, or healthy individuals.

## Methods

### Search strategy

This systematic review was registered with the International Prospective Register of Systematic Reviews (PROSPERO) prior to data extraction (ID: CRD42020186374). The review was conducted in line with the Preferred Reporting Items for Systematic Reviews and Meta-Analyses (PRISMA) statement [16]. Electronic database searches were performed independently by two authors (TC and SL) to identify comparative studies investigating any measure of cutaneous microvascular reactivity in the foot in people with diagnosed diabetes-related Charcot neuroarthropathy of any pattern in either an acute or chronic form from database inception to June 2021 using EMBASE, EBSCO Megafile Ultimate and Cochrane Library. Truncated versions of some search terms were used to ensure that relevant studies were included, and searches were limited to human studies, Table 1. Diabetes was not included as a search term to prevent exclusion of research with sub-analyses in diabetes cohorts or with mixed-group reporting where diabetes-specific data could be requested. This search approach was designed to identify a greater number of articles for screening.

**Table 1:** Search terms of biomedical databases: EMBASE, EBSCO Megafile Ultimate, and Cochrane Library

Group 1	Charcot OR neuroarthropath*
AND	
Group 2	microv* OR cutane* OR skin OR hyperem* OR hyperaem* OR PORH OR PRH OR occlusi* OR iontophoresis OR local heating OR thermal OR warming OR acetylcholine OR ACh OR wavelet OR spectr* OR microdialysis OR Doppler OR LDF OR vasomotion OR capillaroscopy OR TcPO2 or transcutaneous oxygen tension

*PORH and PRH post-occlusive reactive hyperaemia, *LDF* laser-Doppler fluxmetry, *TcPO2* transcutaneous oxygen pressure

### Inclusion/exclusion criteria

The following criteria had to be satisfied for inclusion in the review: published original research investigating cutaneous microvascular reactivity in the feet of adults with diabetes and any pattern of acute or chronic Charcot neuroarthropathy and any non-Charcot adult control group. Included articles required appropriate measures of cutaneous microvascular reactivity using either laser-Doppler fluxmetry, transcutaneous oxygen tension (T_c_PO_2_), or capillaroscopy. Studies only investigating small or large artery (and not microvascular) flow such as with ankle and toe-brachial indices, were excluded. In addition, studies collecting measures of cutaneous microvascular flow that did not assess reactivity or were not conducted in the foot were excluded. For articles where participants with Charcot neuroarthropathy made up a subset of the data and these data were not reported separately, the relevant data were requested from authors and the study was excluded if data were unavailable. Case studies and conference abstracts were excluded where adequate data were not provided in the publications and could not be accessed from authors. Studies investigating other forms of microvascular disease only, such as nephropathy or retinopathy were also ineligible.

### Data collection and analysis

Title, abstract and full-text screen to determine eligibility was performed independently by two authors (TC and SL). Final determination of inclusion by full-text review was conducted in consultation with a third author (VC). Lastly, reference lists of included articles were hand searched to identify any additional potentially relevant articles.

Data extraction was conducted by TC and cross-checked by SL using a customised data collection form with a standard pro forma including publication details (author, year, location), participant characteristics (age, sex, diabetes type and diabetes duration), sample size, measurement technique and outcomes.

### Analyses and meta-analysis

A summary of study results is provided pertaining to various methods of measuring cutaneous microvascular reactivity in people with Charcot neuroarthropathy in context of the relative comparison groups, Table 3. Meta-analysis was performed to investigate the difference in response to thermal hyperaemia in DPN compared to diabetes-related Charcot neuroarthropathy.

Meta-analysis was performed to calculate between-group standardised mean differences with 95% confidence intervals for measures of cutaneous microvascular reactivity in participants with diabetes with and without Charcot neuroarthropathy. The pooled estimate of effect was calculated using a random effects model as it is considered more suitable for combining the results of studies that may not be functionally equivalent [17]. Heterogeneity was then assessed using the Q statistic, *I*^*2*^ and *T*^*2*^. All data analyses were performed using Review Manager (RevMan) Version 5.3 software.

### Methodological quality assessment

The studies that met the inclusion criteria were appraised for risk of bias using a modified Critical Appraisal Skills Programme (CASP) tool and performed independently by two researchers (TC and SL), with disputes arbitrated by a third reviewer (VC).

## Results

**Fig. 1:**
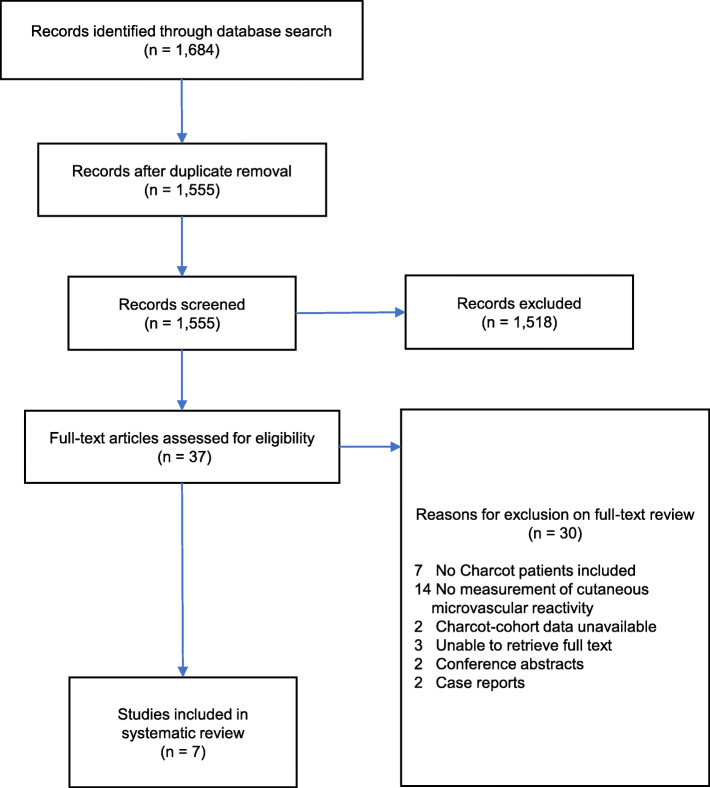
PRISMA flow chart of search strategy

A total of 1,684 articles were retrieved, of which 37 were identified as suitable for full-text review and seven satisfied eligibility for inclusion, Fig. 1. Reason for exclusion based on full-text review were: (i) measures not performed in Charcot neuroarthropathy cohort, (ii) no measurement of cutaneous microvascular reactivity, (iii) Charcot neuroarthropathy cohort data were unavailable or not reported separately and could not be accessed, (iv) full-text unavailable, (v) conference abstracts only, and (vi) case reports.

### Characteristics and overview of included studies

The seven studies that satisfied eligibility for inclusion in this review included a total of 633 participants, with data collected from a combination of one and both lower limbs (*n* = 641 limbs), Table 2. Five studies did not state the number of limbs included [18–22]. Age of participants was reported as either mean (48.3–65.5 years) [19–23] or median with inter-quartile range (IQR) (59 years; IQR = 51–62) [18] and was not reported in one study [24]. Six of the studies specified the number of participants with Type 1 (*n* = 37) and Type 2 (*n* = 112) diabetes [18–23], and one did not [24]. Four studies provided details of sex of participants (*n* = 5–54 males and *n* = 7–16 females) [18, 19, 21, 22] and three did not [20, 23, 24]. Microvascular measures were obtained at the dorsal foot [19, 22–24], plantar hallux [18, 21] and the plantar foot [20]. Studies used either one method or multiple methods of measuring cutaneous microvascular reactivity via laser-Doppler fluxmetry, though in total five used thermal hyperaemia [18, 21–24].

**Table 2:** Participant characteristics

Reference	Number (n)	Sex (M/F)	Age (years)	Diabetes type (type 1 / type 2)	Diabetes duration (years)
Araszkiewicz et al. 2015 [18]	CN: 70 DM: 70	56/14 56/14	CN: median 59 (51–62) DM: median 60 (54–62)	CN: 17/53 DM: 18/52	CN: 16 ± 8 DM: 15 ± 7
Baker et al. 2007 [23]	CN: 13 (4 bilateral; 9 unilateral) CN unaffected foot: 9 DPN: 10 Healthy: 10	NR	CN: 65.5 ± 8.7 DPN: 67.2 ± 7.1 Healthy: 61.4 ± 9.7	CN: 0/13 DPN: 0/10	CN: 20 ± 11.3 CN unaffected foot: 21 ± 10.2 DPN: 19 ± 8.1
Hamdy et al. 2001 [19]	CN: 23 DM: 13 DPN: 33 DPN + PVD: 32 Healthy: 27	CN: 13/10 DM: 8/5 DPN: 24/9 DPN + PVD: 23/9 Healthy: 13/14 Total: 82/46	CN: 57 ± 9 DM: 39 ± 10 DPN: 56 ± 9 DPN + PVD: 60 ± 8 Healthy: 52 ± 13	CN 5/18 DM 9/4 DPN 12/21 DPN + PVD 23/9 Total 41/60	CN: 17 ± 11 DM: 17 ± 7 DPN: 21 ± 12 DPN + PVD: 25 ± 13
Parkhouse et al. 1988 [20]	CN: 8 DM: 14 DPN + ulcer: 11 DM + skin lesions: 9 Healthy: 80	NR	CN: 49.5 ± 13.5 DM: 46.9 ± 12.2 DPN + ulcer: 52.5 ± 8.1 DM + skin lesions: 49.1 ± 12.2 Healthy: 47.2 ± 11.4	Type 1: 14 / Type 2 unclear	Type 1: range (12–57) Other: unclear
Shapiro et al. 1998 [24]	CN: 13 DPN: 12 Healthy: 11	NR	NR	NR	NR
Stevens et al. 1992 [21]	CN (acute): 12 DM: 12 DPN + ulcer: 12 Healthy: 10	CN (acute): 5/7 DM: 6/6 DPN + ulcer: 7/5 Healthy: 5/5 Total: 23/23	CN (acute): median 49.2 (28–69) DM: median 48.3 (32–69) DPN + ulcer: median 51.5 (36–69) Healthy: median 50.1 (31–65)	CN (acute): 10/2 DM: 9/3 DPN + ulcer: 10/2 Total: 29/7	CN (acute): 22.5 ± 12.8 DM: 23.0 ± 14 DPN + ulcer: 24.8 ± 15
Veves et al. 1998 [22]	CN: 23 DM: 13 DPN: 33 DPN + PVD: 32 Healthy: 27	CN: 13/10 DM: 8/5 DPN: 24/9 DPN + PVD: 23/9 Healthy: 13/14 Total: 82/46	CN: 57 ± 9 DM: 39 ± 10 DPN: 56 ± 9 DPN + PVD: 60 ± 8 Healthy: 52 ± 13	CN: 5/18 DM: 9/4 DPN: 12/21 DPN + PVD: 23/9 Total: 41/60	CN: 17 ± 11 DM: 17 ± 7 DPN: 21 ± 12 DPN + PVD: 25 ± 13

*CN* Charcot neuroarthropathy, *DM* uncomplicated diabetes mellitus, *DPN* diabetes-related peripheral neuropathy, *NR* not reported, *PVD* peripheral vascular disease. Age and duration are reported as means ± SD or median (range)

### Cutaneous microvascular reactivity in Charcot neuroarthropathy

Collectively, included studies investigated cutaneous microvascular reactivity in cohorts with Charcot neuroarthropathy compared to healthy controls [19–24], uncomplicated diabetes [18, 19, 21, 22], DPN [19, 21–24], DPN with foot ulceration [21], and DPN with peripheral vascular disease [19, 22], Table 3. As a trend cutaneous microvascular reactivity in Charcot neuroarthropathy groups was impaired compared to both healthy and uncomplicated diabetes groups. In addition, cutaneous microvascular reactivity in the presence of DPN tended to be noticeably worse than in Charcot neuroarthropathy. There were a few deviations from this trend however, such as an impaired response to ACh iontophoresis in a Charcot group compared to DPN group [20], and an exceptionally high response to thermal peak in Charcot versus a healthy group [24].

**Table 3:** Measurement and results of cutaneous microvascular reactivity

Reference	Equipment (site of measure)	Microvascular measures	Charcot group	Comparison group(s)
Araszkiewicz et al. 2015 [18]	Laser-Doppler (plantar hallux / non-glabrous)	Thermal peak – Median (IQR) Post-occlusive Reactive Hyperaemia – Median (IQR)	156 (93–240) 142.5 (98–218)	DM: 238 (155–300) DM: 143 (98–222)
Baker et al. 2007 [23]	Laser-Doppler (dorsal foot / glabrous)	Thermal peak – Mean ± SD	CN: 432 ± 88 CN contralateral foot: 417 ± 110	DPN: 262 ± 71 Healthy: 594 ± 94
Hamdy et al. 2001 [19]	Laser-Doppler (dorsal foot / glabrous)	ACh Iontophoresis – Median (25th – 75th quartiles)	227 (86–554)	DM: 578 (152–1858) DPN: 90 (15–378) DPN and PVD: 74 (1-212) Healthy: 411 (148–641)
Parkhouse et al. 1988 [20]	Laser-Doppler (plantar foot / non-glabrous)	ACh Iontophoresis – Mean	3.0	Type 1 DM: 12.5 DPN + ulcers: 4.7 DM and skin lesions: 5.8 Healthy: 11.5
Shapiro et al. 1998 [24]	Laser-Doppler (dorsal foot / glabrous)	Thermal AuC – Mean ± SD Thermal vasomotion – Mean ± SD	64.8 ± 56.9 968.2 ± 450.2	DPN: 6.6 ± 1.7 Healthy: 12.9 ± 5.3 DPN: 326.6 ± 176.4 Healthy: 1162.5 ± 279.7
Stevens et al. 1992 [21]*	Laser-Doppler (plantar hallux / non-glabrous)	Thermal peak – Mean ± SD	Acute Charcot 41.0 ± 19.2 CN contralateral foot: 63.4 ± 28.7	DM: 62.7 ± 47 DPN + ulcers: 28.9 ± 37.4 Healthy: 76.3 ± 33.9
Veves et al. 1998 [22]	Microspan TcPO_2_ meter (Dorsal foot / glabrous) Laser-Doppler (dorsal foot)	Thermal peak – Median (IQR) Post-iontophoresis – Median (IQR)	94 (57–120) 0.3 (0.2–0.4)	DM: 119 (76–175) DPN: 71 (35–84) DPN + PVD: 47 (26–64) Healthy: 127 (99–162) DM: 0.5 (0.4–0.7) DPN: 0.3 (0.3–0.4) DPN + PVD: 0.3 (0.3–0.4) Healthy: 0.5 (0.3–0.7)

IQR, inter-quartile range; DM, uncomplicated diabetes mellitus; CN, Charcot neuroarthropathy; DPN, diabetes-related peripheral neuropathy; PVD, peripheral vascular disease; AuC, area under the curve; TcPO_2_, transcutaneous oxygen tension

### Meta-analysis

**Fig. 2:**
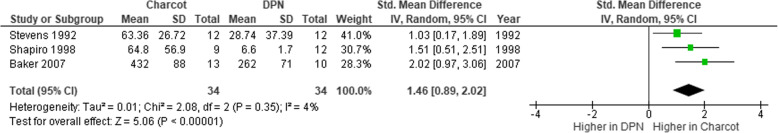
Forest plot of cutaneous microvascular reactivity to thermal hyperaemia in Charcot neuroarthropathy and DPN

Three studies used laser-Doppler fluxmetry to compare the peak response to local thermal stimulus between Charcot neuroarthropathy and DPN groups and provided adequate data for meta-analysis, Fig. 2. In the case of Stevens et al., [21] due to the acute Charcot presentation resulting in heightened inflammatory response, the data for the contralateral limb were included in the meta-analysis. The meta-analysis demonstrated a significant difference in peak response to thermal hyperaemia between Charcot neuroarthropathy and DPN with a large, pooled effect size (SMD 1.46 95% CI: 0.89–2.02) and low heterogeneity (*I*^*2*^ = 4%, *T*^*2*^ *=* 0.01) indicating that cutaneous microvascular reactivity is more impaired in DPN than in Charcot neuroarthropathy.

### Critical appraisal of the included articles

All studies appeared to address a clearly focused topic and measured the exposures and outcomes accurately to minimise the bias and to provide reliable results, Table 4. Some considerations from this appraisal include potential issues surrounding participant recruitment [20, 21, 24], and ability to identify all relevant confounding factors in the design, analysis or otherwise [20–22]. All studies considered a range of factors affecting reactivity when undertaking measurements. Pre-test rest was reported, ranging from 10 to 30 min [18–21, 24], with room acclimatised to between 22 and 26 °C [19–22]. Other potential vasoactive influencers such as caffeine, nicotine or physical activity were only clearly stated in two studies, where fasting from 30 to 120 min prior to measurements was specifically mentioned [18, 21].

**Table 4:** Methodological quality appraisal using a modified Critical Appraisal Skills Programme (CASP) checklist

Item	Araszkiewicz et al. (2015)	Baker et al. (2007)	Hamdy et al. (2001)	Parkhouse et al. (1988)	Shapiro et al. (1998)	Stevens et al. (1992)	Veves et al. (1998)
1. Did the study address a clearly focused issue?	Π	Π	Π	Π	Π	Π	Π
2. Was the cohort recruited in an acceptable way?	Π	Π	Π	?	?	?	Π
3. Was the exposure accurately measured to minimise bias?	Π	Π	Π	Π	Π	Π	Π
4. Was the outcome accurately measured to minimise bias?	Π	Π	Π	Π	Π	Π	Π
5. Have the authors addressed confounding factors in the study design and/or analysis?	Π	Π	Π	?	Π	?	?
6. Are the results of the study clearly presented?	Π	Π	Π	?	Π	?	Π
7. Are the results precise?	Π	Π	Π	Π	Π	Π	Π
8. Do the results reflect a validated model?	Π	Π	Π	Π	Π	Π	Π

Π: yes; ?: not sure; Ο: no

## Discussion

The aim of this review was to systematically evaluate the literature comparing the relationship between cutaneous microvascular reactivity in the foot of adults with diabetes-related Charcot neuroarthropathy and other patient phenotypes including healthy adults [19–23] and those with diabetes [18–22] or DPN [19, 20, 22–24] only. The findings of this review are that the cutaneous microvascular reactivity in people with Charcot neuroarthropathy as determined by microvascular response to stimulus, is impaired compared to adults with diabetes alone. However, most notably, we demonstrated by meta-analysis that in people with DPN only, the cutaneous microvascular response is significantly more impaired than in those with Charcot neuroarthropathy. This supports the potential for there to be altered vascular control resulting in blood flow in the foot in individuals who develop Charcot neuroarthropathy that is greater than that found more broadly in DPN cohorts.

Beyond the results of the meta-analysis, the overall results of this review suggest cutaneous microvascular reactivity in those with Charcot neuroarthropathy sits between people with diabetes alone and the impairment of cutaneous microvascular reactivity seen in the presence of DPN. That is, the microvascular reactivity in the Charcot neuroarthropathy groups is better than those in the DPN groups but worse than that of diabetes groups without foot complications. While these broader findings are also consistent with vascular theory relating to the development of Charcot neuroarthropathy, these findings remain in the context of cutaneous microvascular reactivity. Key to theoretical links between altered vascular function and the development of Charcot neuroarthropathy is altered blood flow to bone. While bone relies on blood supply form capillary networks in bone marrow, due to the difficulty with measuring blood flow to bone, there has been limited investigation of changes related to the presence of DPN [25]. Therefore, the results of studies included in this review assessing peripheral cutaneous microvascular reactivity may not be representative of blood flow to bone and may not have a direct role in the pathogenesis of Charcot neuroarthropathy. Similarly, six of the seven studies included in this review recruited Charcot neuroarthropathy cohorts in the chronic phase of the disease process [18–20, 22–24]. Therefore, the differences identified between the groups with diabetes and DPN alone and groups with chronic Charcot neuroarthropathy may have been the result of the condition, rather than related to its development. However, Baker et al., found the same neurovascular abnormalities in affected limbs and contralateral limbs unaffected by Charcot neuroarthropathy – albeit in the quiescent phase - suggesting that impaired cutaneous microvascular reactivity may precede Charcot neuroarthropathy [23]. A better understanding of this pathophysiology could identify if measures of cutaneous microvascular reactivity have diagnostic potential for Charcot neuroarthropathy.

The results of this systematic review and meta-analysis highlight the need for further research investigating mechanisms for the differences observed in cutaneous microvascular function in those with Charcot neuroarthropathy and those with DPN. Previous research has demonstrated that response to heat, controlled by small diameter nerve fibres, remains intact in those with Charcot neuroarthropathy but is reduced in those with DPN alone [22]. This is likely to contribute to the differences in microvascular function seen between DPN and Charcot groups in response to thermal hyperaemia. Damage to these fibres has been shown to occur independent of large fibre neuropathy where there is loss of pressure and vibration perception however clinically, small fibre neuropathy is less frequently tested for [26]. As small nerve fibre dysfunction has been proposed to result in loss of capillary flow due to arterio-venous shunting, retaining normal function of these fibres would be consistent with the increased cutaneous microvascular blood flow found in those with Charcot [27, 28]. While large fibre neuropathy is common to both DPN and Charcot neuroarthropathy, testing small fibre nerve function may offer an additional mechanism of identifying those at risk of developing the condition.

The results of this systematic review and meta-analysis have identified likely differences in cutaneous microvascular reactivity in diabetes cohorts with DPN and with and without Charcot neuroarthropathy. The apparent ameliorated effect of DPN on cutaneous microvascular reactivity in those with Charcot neuroarthropathy suggests that this may be related to the pathological process of the condition. In light of this, further large-scale prospective investigations are required that include comprehensive baseline measures and ongoing assessment of cutaneous microvascular reactivity and DPN by fibre type. Furthermore, the possibility of more sensitive and specific testing for DPN in clinical practice may offer an additional method to more accurately profile risk status of patients with diabetes for the condition. In addition, it should be noted that equipment used in the included studies is generally not available for clinical use due to expense, expertise required and associated time constraints. Therefore, though these measures aid in understanding pathogenesis and diagnosis, alternate clinical methods are required for widespread adaptation and therefore the direct transfer of the results of these studies has implications for wider use and interpretation. This highlights the need for further investigations to establish the relationship between cutaneous microvascular function and small arterial function so that surrogate measurement, which is readily available at many clinics (e.g. photoplethysmography), could be used as a screening tool.

### Limitations

Though being rigorous, chances are that our search strategy would miss some potentially relevant papers. Of note there is currently no consensus or guidelines to specify what constitutes pathological values or the degree of severity when it comes to assessing cutaneous microvascular reactivity. Thus, the articles included in this review were required to compare cutaneous microvascular reactivity in Charcot neuroarthropathy to other groups such as diabetes without complications or diabetes with ulcers, for example. Although there is no guideline on the pathological values, both diminished response in post-occlusive reactive hyperaemia and thermal hyperaemia are considered directionally pathological [29–31]. However, if used for diagnostic purposes, these two tests would need to be performed and due to the inconvenience and expense of the equipment, it is not readily available at usual clinics. In addition, this review does not provide a conclusion regarding the cause of Charcot neuroarthropathy. The meta-analysis that we performed could only include a small number of studies due to lacking appropriate data. Though we observed a higher peak response to local thermal stimulus which is statistically significant in the meta-analysis, this could not explain the underlying reason. Additionally, our meta-analysis included two studies assessing the affected foot of people with a non-acute Charcot presentation [23, 24] and one study assessing the unaffected foot in people with an acute presentation [21]. The analysis therefore needs to be considered in this context. The seven included studies contained different methodologies and equipment making it difficult to compare different cohorts on the same ground to work out a pathological value that could be applied as a guideline.

## Conclusions

This review suggests that cutaneous microvascular reactivity in the foot is impaired in the presence of both Charcot neuroarthropathy and DPN compared to that in people with uncomplicated diabetes. Though DPN is seen as a precursor to Charcot neuroarthropathy, microvascular reactivity appears to be further impaired in DPN compared to diabetes-related Charcot neuroarthropathy. It is unknown if this occurs prior to, or as a result of, Charcot neuroarthropathy, with limited evidence suggesting the former [23]. However, this review suggests a potential for altered vascular control involving a relative increase in blood flow specific to individuals who develop Charcot neuroarthropathy that is not reflected more broadly in DPN cohorts. These findings support the need for future research examining the role of nerve fibre type in microvascular function in the presence of DPN. Further investigation is warranted to determine the role of cutaneous microvascular dysfunction in the pathogenesis of Charcot neuroarthropathy.

## Data Availability

The datasets used and/or analysed during the current study are available from the corresponding author on reasonable request.

## Abbreviations

ACh: acetylcholine
CASP: Critical Appraisal Skills Programme
DPN: diabetes-related peripheral neuropathy
